# PEACH, a smartphone- and conversational agent-based coaching intervention for intentional personality change: study protocol of a randomized, wait-list controlled trial

**DOI:** 10.1186/s40359-018-0257-9

**Published:** 2018-09-04

**Authors:** Mirjam Stieger, Marcia Nißen, Dominik Rüegger, Tobias Kowatsch, Christoph Flückiger, Mathias Allemand

**Affiliations:** 1 Department of Psychology and URPP Dynamics of Healthy Aging, University of Zurich, Andreasstrasse 15, 8050 Zürich, Switzerland; 2 Technology Marketing, ETH Zurich, Weinbergstrasse 56/58, 8092 Zürich, Switzerland; 3 Center for Digital Health Interventions, Information Management, ETH Zurich, Weinbergstrasse 56/58, 8092 Zürich, Switzerland; 4 Center for Digital Health Interventions, Institute of Technology Management, University of St.Gallen (ITEM-HSG), Dufourstrasse 40a, St.Gallen, Switzerland; 5 Department of Psychology, University of Zurich, Binzmühlestrasse 14/04, 8050 Zürich, Switzerland

**Keywords:** Intentional personality change, personality change intervention, coaching intervention, smartphone, conversational agent

## Abstract

**Background:**

This protocol describes a study that will test the effectiveness of a 10-week non-clinical psychological coaching intervention for intentional personality change using a smartphone application. The goal of the intervention is to coach individuals who are willing and motivated to change some aspects of their personality, i.e., the Big Five personality traits. The intervention is based on empirically derived general change mechanisms from psychotherapy process-outcome research. It uses the smartphone application PEACH (PErsonality coACH) to allow for a scalable assessment and tailored interventions in the everyday life of participants. A conversational agent will be used as a digital coach to support participants to achieve their personality change goals. The goal of the study is to examine the effectiveness of the intervention at post-test assessment and three-month follow-up.

**Methods/Design:**

A 2x2 factorial between-subject randomized, wait-list controlled trial with intensive longitudinal methods will be conducted to examine the effectiveness of the intervention. Participants will be randomized to one of four conditions. One experimental condition includes a conversational agent with high self-awareness to deliver the coaching program. The other experimental condition includes a conversational agent with low self-awareness. Two wait-list conditions refer to the same two experimental conditions, albeit with four weeks without intervention at the beginning of the study. The 10-week intervention includes different types of micro-interventions: (a) individualized implementation intentions, (b) psychoeducation, (c) behavioral activation tasks, (d) self-reflection, (e) resource activation, and (f) individualized progress feedback. Study participants will be at least 900 German-speaking adults (18 years and older) who install the PEACH application on their smartphones, give their informed consent, pass the screening assessment, take part in the pre-test assessment and are motivated to change or modify some aspects of their personality.

**Discussion:**

This is the first study testing the effectiveness of a smartphone- and conversational agent-based coaching intervention for intended personality change. Given that this novel intervention approach proves effective, it could be implemented in various non-clinical settings and could reach large numbers of people due to its low-threshold character and technical scalability.

## Background

There is a recent debate in personality science whether and how personality traits can be intentionally modified or changed over short periods of time by intervention efforts. Although available research suggests that most people want to change or modify some aspects of their personality [1–3], psychological interventions for intentional personality change are almost lacking. Only a few studies have examined intentional personality change over shorter periods of time [4–6]. These very few existing studies are promising and suggest that intended trait change in a desired direction is possible. However, it is still an open question whether personality change can be maintained or rather reflects temporary changes that revert over time. This protocol describes a study that will test the effectiveness of a non-clinical psychological coaching intervention for intentional personality change that focuses on the Big Five personality traits, i.e., neuroticism, extraversion, openness to experience, agreeableness, and conscientiousness.

## Conceptual Framework of the Intervention

Since intervention efforts for intended personality change are in their infancy, conceptual frameworks are needed to develop theory-driven intervention programs. One approach would be to carefully develop specified treatments/treatment guidelines for changing particular personality traits. The other approach would be to develop interventions based on more general (common) intervention principles [7]. The present coaching intervention is based on a general (common) change mechanisms intervention framework. General change mechanisms are assumed to be responsible for intermediate changes in someone’s characteristics, skills, experiences, and behaviors, and eventually lead to improvements in the ultimate outcome or targeted goal of an intervention. Allemand and Flückiger [7] argue that four empirically derived general change mechanisms from psychotherapy process-outcome research [8–11] provide useful heuristic principles for intentional personality change interventions and help to maximize the effectiveness of intervention efforts. The four mechanisms are: (1) actuating discrepancy awareness, (2) targeting thoughts and feelings (insight), (3) targeting behaviors (practice), and (4) activating strengths and resources. These mechanisms highlight different perspectives of the immediate individual psychological outcomes and are highly connected with each other [12]. In order to target those general change mechanisms and to promote the change process, the coaching intervention includes several micro-interventions. Micro-interventions (specific tools and techniques) are small interventions that are essential in helping individuals to modify or change trait-related experiences and behaviors in concrete real-life situations and help to maintain the change process [13].

### Actuating discrepancy awareness

The first change mechanism focuses on the awareness of differences between the actual and the desired personality, which might facilitate the change process. The idea is that personality traits can be most effectively targeted and altered while people explore potential gaps between their actual and desired personality (cf. [14]). Examples of micro-interventions that target this change mechanism are (a) the motivational interviewing approach, (b) miracle questions, and (c) individualized progress feedback. The *motivational interviewing approach* [15] serves to counterbalance advantages and disadvantages of change and might eventually enhance individual change motivation. By writing down pros and cons of the actual and desired behavior and experience, people can evaluate the gap between their actual and desired personality. *Miracle questions* help people to think about their future goals and their desired personality and thus actuate discrepancy awareness between the actual and the desired personality. Miracle questions are basically thought experiments, which ask people to imagine their desired future and personality [16]. Individually tailored *progress feedback* is one of the most commonly used change techniques in smartphone-based health interventions [13] that helps people to focus on their discrepancy awareness.

### Targeting thoughts and feelings to realize insight

The second mechanism emphasizes reflective processes, which may promote the personality change process by helping individuals to reflect their thoughts, feelings, and behaviors in a more systematic way. The following five micro-interventions are known to be effective to activate this change mechanism: (a) systematic reflection, (b) psychoeducation, (c) observational learning, (d) introspection, and (e) identification of situational/contextual triggers. *Systematic reflection* is a micro-intervention that helps people to learn from experiences including failures and successes [17]. Changing aspects of one’s personality is hard and is related to experiences of failures. Systematic reflection helps to focus on the goal rather than on emotional reactions after a failed task. To promote the change process, it is also important to understand own beliefs and expectations. Since people may have different self-theories about the changeability of different aspects of personality [18], fostering the knowledge transfer about personality change in the form of *psychoeducation* may further promote the change process. Psychoeducation is a prominent tool in cognitive behavioral therapy [19]. Other micro-interventions, which also target thoughts and feelings, are the observation and modeling of others’ behaviors (*observational learning*) [20, 21], watching one’s own behaviors, thoughts, and feelings (*introspection*) [21, 22], and identifying *situational and contextual triggers* (e.g., people, society, surroundings; [23]). Being aware of situational and contextual triggers, which are connected to the desired or actual behavior, can actually help to show desired behaviors more often and to avoid actual behaviors [23].

### Targeting behaviors to realize practice

This mechanism focuses on learning and reinforcing new behaviors and skills, such as compensatory or coping skills, and to behave in new roles. To achieve change goals, individuals should gradually increase engagement in activities and new behaviors connected to their change goals. Two micro-interventions are included in the intervention to target this change mechanism: (a) implementation intentions and (b) behavioral activation. Generating *implementation intentions* in the form of specific “if-then plans” can lead to better goal attainment and help individuals in habit formation [24]. This micro-intervention was successfully used in previous intervention work for intentional personality change [5]. *Behavioral activation tasks* help individuals to perform novel behaviors and activities. Behavioral activation is based on principles of reinforcement and learning theory and was originally developed for the treatment of depression [25]. Magidson and colleagues’ [26] suggest this micro-intervention also for intentional personality interventions and used it in their case study.

### Activate strengths and resources to realize strengths-orientation

This change mechanism capitalizes on individual and interpersonal strengths and resources. Resources might be related to personal skills and capabilities, motivational readiness and preparedness for change, as well as social support. Micro-interventions identified to target this mechanism include (a) organizing a change team, (b) keeping a diary of strengths and resources, (c) using the tree of resources, and (d) thinking about future plans, dreams and hopes. An informed *change team*, including significant others such as friends and family members can provide social support throughout an intervention and help people to attain their change goals [27]. Keeping a *diary of strengths and resources* [28] or to write down individual resources inside the *tree of resources* [8, 29] can further promote the change process by reflecting about personal strengths and positive aspects of life. Another micro-intervention activates individuals’ resources and enhances change motivation by *thinking about future plans, dreams and hopes* by getting asked questions derived from the life story interview approach [30].

## Smartphone-Based Coaching Interventions

Smartphones provide a powerful tool set for psychological and behavioral micro-interventions for several reasons [31–38]. First, smartphones are ubiquitous with increasingly powerful technical abilities and make sophisticated micro-interventions appealing and widely applicable. Second, unlike desktop computers, laptops or tablets, smartphones are nearly always with the person. Third, people often have a positive emotional attachment to and daily routines in dealing with their smartphones, which can reduce the barriers to adoption and increase acceptance of micro-interventions. Fourth, the combination of powerful technical abilities of smartphones and their proximity to their owners offers the ability to detect useful context information that can be used to individualize interventions. Moreover, context awareness features enabled through sensing and phone-based personal information allows creating just-in-time micro-interventions that provide users with support at times when that support is most needed. Finally, interventions using smartphones are scalable, cost-effective, low-threshold, applicable to a wide variety of participants and show promising retention rates. For example, a recent study in the public health context found that owning a smartphone “was not a major barrier to study participation for most respondents [...] including those who were unemployed”, i.e. with a low socio economic status [39]. In another recent study, retention rates of smartphone-based interventions are promising as participants had eight conversational turns with a smartphone-based chatbot per day on average over the course of six months [40, 41].

### The talk-and-tools-paradigm

Smartphone interfaces also enable the application of the so-called talk-and-tools paradigm [42, 43]. That is, smartphones are able to offer scalable communication features with the help of conversational agents (the *“Talk”*, e.g., for motivational interviewing purposes), i.e., computer programs that imitate a conversation with a human being [44–47]. In contrast to popular voice-based conversational agents such as Amazon’s Alexa, Apple’s Siri, text-based conversational agents (often called “chatbots”) are so far less prominent. Promising examples include Florence (getflorence.co.uk), Lark (web.lark.com) or Woebot (woebot.io). In contrast, interfaces of smartphones can also be used to deliver a broad range of *“Tools”*, i.e., the building blocks of micro-interventions (e.g., keeping a diary of resources, a reminder for individual implementation intentions or the delivery of psychoeducation video clips). The application of this talk-and-tools paradigm can not only complement and extend existing face-to-face counseling sessions to the everyday life of individuals, but it can also provide new means to offer smartphone-based coaching interventions in a scalable fashion where a personal coaching approach is not feasible due to limited reach, personnel or budget.

### Design of conversational agents

Due to limited evidence on effective designs of text-based conversational agents on mobile devices [48, 49], it is essential to study design features of conversational agents and how they help individuals to reach their goals. Conversational agents are designed to interact with a human – like a human. The *Computers as Social Actors* theorem by Reeves and Nass [50] confirms that individuals apply social behaviors and heuristics typical for social interactions with other human beings to interactions with computers – and conversational agents.

Research in the field of counselling psychology and psychotherapy has shown that *working alliance,* a collaborative quality and the degree to which health professionals and patients engage with each other, is associated with the therapeutic process and robustly linked to treatment success in face-to-face therapy as well as in online therapy (*r* = .28; [51]) [52, 53]. The concept of working alliance can be adapted to the “relationship” between individuals and conversational agents and their interactions (e.g., quality and length of messages exchanged or frequency of interactions). It can be expected, that when a conversational agent takes over the role of a communication partner and embodies a digital coach, its communication style and role will affect relationship-building processes and, in part, treatment success (e.g., [54, 55]).

Hence, it can be assumed that the choice of specific verbal cues will increase an individual’s working alliance with a conversational agent. The present conversational agent-based intervention will focus on one specific verbal cue, namely whether the chatbot can refer to itself using the first-person pronoun “I”. The use of “I” automatically implies a sense of human self-awareness or self-concept by the chatbot [56], making it more anthropomorphic and relatable, than a conversational agent without a self-concept.

In order to test the effects of a self-aware versus a non-self-aware conversational agent on working alliance and intervention effectiveness, two conversational agents will be experimentally manipulated, such that a “self-aware” conversational agent will present itself as a tangible and present entity by actively referring to itself (“May I help you?”) in contrast to an impersonal control conversational agent which will refrain from referring to itself (“Do you need help?”) and remains less tangible as an entity, fading the anthropomorphic identity of the conversational agent into the background. The overall conversational streams, message lengths, coaching elements, and schedule will be kept the same in both conditions.

## Research Goals and Hypotheses

The first goal of the present study is to examine the effectiveness of PEACH, a smartphone- and conversational agent-based coaching intervention for intentional personality change. The outcome research hypothesis is that two experimental conditions (high versus low self-aware conversational agent) will be more effective with respect to personality trait change in comparison to the two waiting list conditions. Furthermore, based on previous work on the effects of anthropomorphized computer-mediated communication on human behavior [57], the differential outcome research hypothesis is that the self-aware conversational agent will be more effective in terms of relationship-building, promoting intervention adherence and thus treatment success than the low self-aware conversational agent.

The second goal is to explore underlying processes and mechanisms that improve the outcomes of the intervention. Two approaches are used for process assessments: self-reports and smartphone sensing. Both methods include an intensive longitudinal design. This allows exploring associations between actively (self-reports) and passively (sensors) assessed intervention processes.

## Methods/Design

### Design

In this study protocol, we describe a 2x2 factorial between-subject randomized, wait-list controlled trial with intensive longitudinal methods studying the effectiveness of a 10-week smartphone- and conversational agent-based coaching intervention for intentional personality change. The effectiveness of the intervention will be compared across two dimensions: intervention (experimental versus wait-list control) and conversational agent design (high versus low self-awareness). Participants will be randomly assigned to one of four conditions: (a) experimental condition 1: conversational agent with high self-awareness, (b) experimental condition 2: conversational agent with low self-awareness, (c) wait-list condition 1: conversational agent with high self-awareness, (d) wait-list condition 2: conversational agent with low self-awareness. Participants in the wait-list control conditions will receive no intervention for the first four weeks to document the natural course of their personality change without expecting interventional effects. To monitor progress, the wait-list control groups will respond to the same weekly questionnaires during those four weeks as the subjects from the experimental conditions. Additionally, they are passively tracked by smartphone sensors. After the four weeks without any intervention, subjects of the wait-list control conditions will receive the same intervention as subjects of the experimental conditions - depending on their conversational agent embedding high or low self-awareness cues.

### Participants and Recruitment

The targeted sample will include at least 900 German-speaking adults, who install the PEACH App on their smartphones, give informed consent, pass the screening, fill in the pre-test assessment and start with the intervention. To assure an adequate power to detect statistical significance and to demonstrate a small to medium effect of a pre-post time by group interaction we require data from 300 participants. Assuming an α error level of 0.05, a statistical power (1-β) of 0.80, and a correlation of 0.40 between the pre- and post-measurements and 75 completers for each group, we would be able to detect a small effect of Cohen’s *d* = .22. Computing power for repeated measures, which is the case in this study, is more complex. As such, this power analysis only gives a rough idea of the effect sizes the study could reasonably detect. In a similar study [33], 67% of participants completed the post-test survey after 6 weeks, from a cohort of 273 who started the intervention. Based on this estimate and taking the longer duration of this study into account, we expect even more attrition. Should drop-out rates be higher than expected, we may recruit additional participants to ensure sufficient statistical power. To be eligible for the study, participants must be: (1) 18 years or older; (2) able to read German; (3) not in a psychotherapeutic or psychiatric treatment; (4) owner of a smartphone (Android or iOS) with mobile internet connection; and (5) interested and motivated to participate at the intervention and to change some aspects of their personality. The focus of this intervention study is explicitly on healthy adults. Thus, adults with mental health disorders and other psychosocial problems will be excluded. Participants will complete an online eligibility screening that checks for the inclusion criteria. Excluded candidates with mental health disorders and psychosocial problems will be provided with an information and contact details of the psychological counseling service of the University of Zurich.

**Fig. 1:**
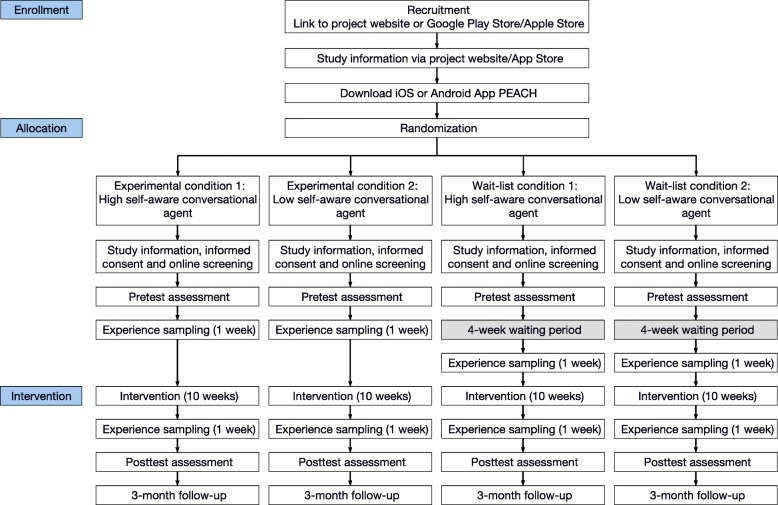
Study design

We will primarily use university mailings and social media advertisements for the recruitment process. Additionally, potential participants will respond to flyers or word-of-mouth recruitment. Interested people will be directed to either the website of the project (www.personalitycoach.ch) or to the Apple Store/Google Play Store to receive detailed information about the study aims, interventions, assessments, reimbursement, and data protection and download the mobile application. Participants will be automatically and randomly assigned to one of four conditions (Fig. 1). In total, the two experimental conditions will be oversampled and will include 2/3 and the control condition 1/3 of all participants (full randomization in all four conditions). The automated allocation and randomization procedures will be computer generated. In this way, we aim to ensure that the conditions are fully randomized with respect to the participants’ baseline characteristics (allocation concealment). Because all participants will be treated using a comparable coaching intervention, participants are blinded to the two conversational agents. Spill-over effects could occur since participants might know each other and talk about the procedure of the intervention. After obtaining informed consent and passing the screening assessment, participants will be directed to the pre-test assessment. The procedure and design of the study are also depicted in Fig. 1.

Reimbursement of 25 Swiss Francs for taking part in the pre-test and follow-up assessment will be offered to study participants. Consistent with prior work (e.g. [58]), participants will be able to earn credits for active participation and by fulfilling specific tasks during the intervention such as engaging with the conversational agent (maximum 8 credits per day), experience sampling measurements (3 credits per measurement occasion), weekly assessments (20 credits per assessment), and photo uploads (15 credits per upload). Participants can collect 1,000 credits in total and reach bronze status with 250 credits or more, silver status with 500 credits or more, and gold status with 750 credits or more. According to their status, participants earn tickets for the lottery (bronze status = 1 ticket, silver status = 5 tickets, gold status = 10 tickets). Participants can win 100 Swiss francs, 200 Swiss francs, and 300 Swiss francs in cash.

### Procedure

The procedure is shown in Fig. 1. After having completed the pre-test assessment, participants get instant feedback on their actual Big Five personality trait profile (BFI-2; [59]). This feedback should help participants to choose their appropriate change goal. Participants have to pick one change profile out of nine, which fits the most to their individual change goal. Each of these nine profiles explains normal characteristics of a person with high or low levels in the corresponding Big Five personality trait. To be more precise, participants can choose between nine *personality change profiles*: (1) increase in conscientiousness, (2) decrease in conscientiousness, (3) increase in extraversion, (4) decrease in extraversion, (5) increase in open-mindedness, (6) decrease in open-mindedness, (7) increase in agreeableness, (8) decrease in agreeableness, and (9) decrease in negative emotionality. For ethical reasons an intervention to increase negative emotionality will not be offered. Participant then indicate the strength of their chosen change goal on an 8-point scale from 0 = *not at all* to 7 = *totally* and their willingness to change (i.e., goal commitment and goal attainability; [60]). Additionally, participants are asked to share a link with at least three close friends, family members and their intimate partner to obtain an observer-report on the Big Five personality traits (BFI-2-S; [61]) (Table 2).

**Fig. 2:**
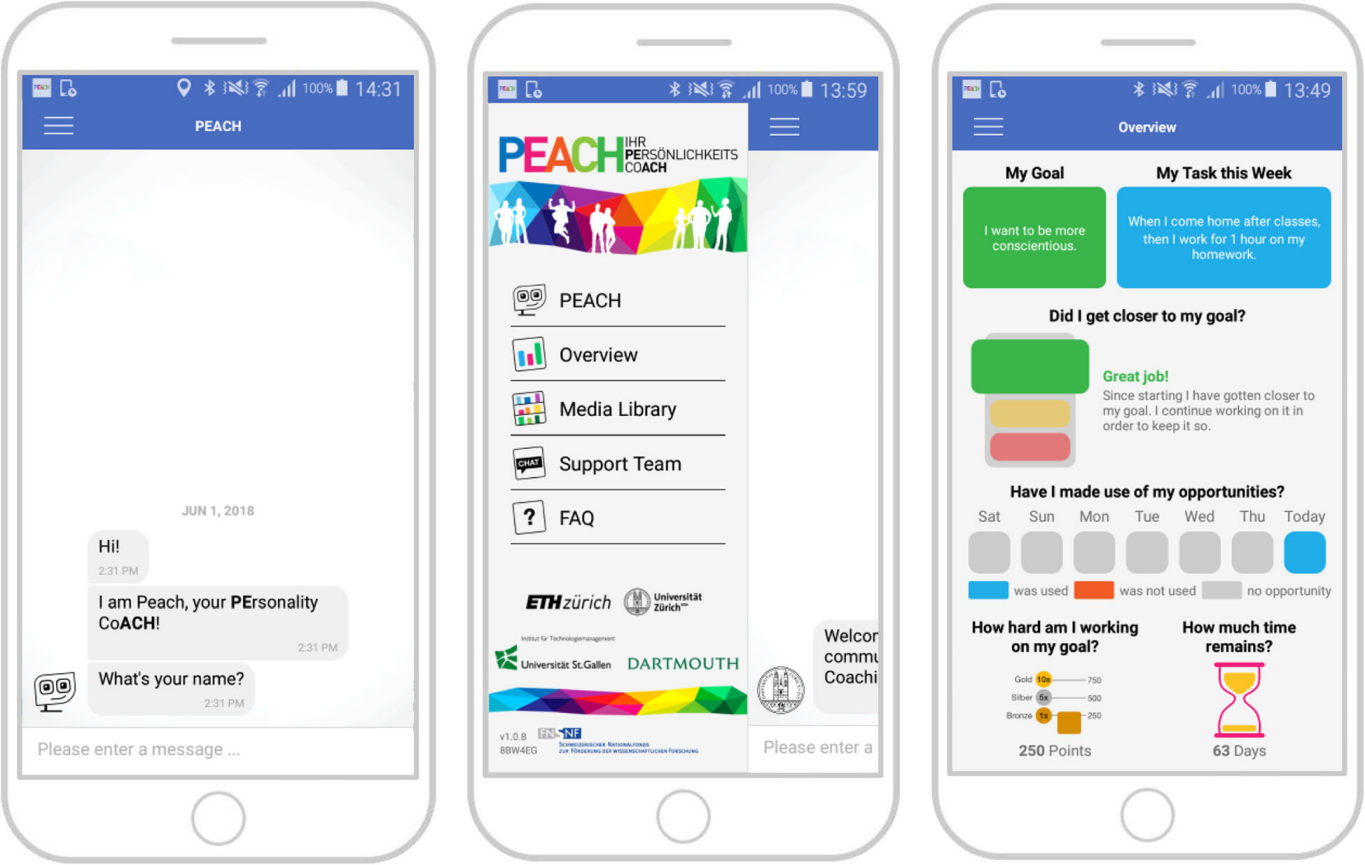
The PEACH App and its Components. *Note.* Chat-based interaction with the conversational agent PEACH (left), the sidebar (middle) that allows participants to switch to either a dashboard with a personalized overview of the current status of the intervention (right), a media library used for psychoeducational video clips, a chat channel that allows participants to communicate with the “Support-Team”, or a page for frequently asked questions about the PEACH study and the app

The first week of the study is an experience sampling week to measure personality manifestations in daily life (for more details, see below). The personality change intervention then lasts over 10 weeks. For each of the 10 weeks, weekly core themes will be provided (Table 1). Moreover, six different types of micro-interventions will be used in the intervention (see below). All participants are actively involved in two daily dialogues with the conversational agent. In the morning at an individually preferred time participants receive the first message for the morning dialogue and in the evening again at an individually preferred time participants receive the first message for the evening dialogue. Participants have the opportunity to read the dialogue until it is time for the next dialogue. A conversational agent will be used to remind participants to complete questionnaires, to guide them through micro-interventions, to promote commitment, to motivate participants, and to support the change process (Fig. 2). During these conversations, a combination of pre-defined answers and free-text input is used to constrain the dialog along pre-defined counselling paths and to give participants autonomy where needed (e.g., for the definition of implementation intentions in the if-then form). If participants do not actively use the PEACH app over three days, the study team will contact them via the “Support-Team Channel” (Fig. 2) and ask them whether there occurred any problems or whether they have any unanswered questions to promote adherence. After the intervention, there is a second experience sampling week and then participants are asked to answer the post-test assessment and the three-month follow-up assessment (Fig. 1). Moreover, participants were asked at post-test and follow-up assessment to share a link with their close friends, family members or intimate partners, who already provided their observer-reports at pre-test assessment, to obtain a second and third observer-report on the Big Five personality traits (BFI-S-S; [61]).

**Table 1:** Schedule of weekly core themes and micro-interventions

Week	Weekly core theme (Source)	Brief description	Individualized implementation intention^c^	Psycho-education^b^	Behavioral activation tasks^c^	Individualized progress feedback^a^
1	Organizing a change team^d^ [27]	Participants are asked to inform 1-3 significant others such as friends or family members to talk with them about their change goals, the coaching intervention itself and to keep them updated during the intervention.	Implementation intention 1	Daily film clip or scientific input	Behavioral activation task 1	Dashboard
2	Learning from experiences by systematic reflection^b^ [17]	People are asked to analyze their own behavior and advance explanations for the resulting success or failure to learn from both. Questions that prompt self-explanations include: “How did you contribute to the performance?” or “How effective were you in the experience”. Then participants are confronted with questions such as “Consider a different approach that could have been taken.” And finally they should ask themselves: “What worked and what did not work? How will you behave in the future?”	Implementation intention 2	Daily film clip or scientific input	Behavioral activation task 2	Dashboard
3	Identifying situational/ contextual triggers^b^ [23]	Participants learn how to identify situational and contextual triggers (e.g., people, places, time in the day) that help or hinder them to show their desired behavior.	Implementation intention 3	Daily film clip or scientific input	Behavioral activation task 3	Dashboard
4	Thinking and writing about the pro’s and con’s of change^a^ [15]	Participants think about advantages and disadvantages of changing in the desired direction and of staying the same. This might eventually also enhance individual change motivation.	Implementation intention 4	Daily film clip or scientific input	Behavioral activation task 4	Dashboard
5	Learning from others by observational learning^b^ [20, 21]	Participants should look out for people in their environment, who already show their desired behavior. They analyze what these people are doing differently and try to model this behavior.	Implementation intention 5	Daily film clip or scientific input	Behavioral activation task 5	Dashboard
6	Self-reflection by means of introspection^b^ [21, 22]	Participants should watch their own thoughts and feelings when they are able to show their desired behavior and thoughts and feelings when they are not able to show the desired behavior.	Implementation intention 6	Daily film clip or scientific input	Behavioral activation task 6	Dashboard
7	Keeping a diary of strengths and resources^d^ [28]	Participants are asked to think about what they are grateful in life and about their personal strengths.	Implementation intention 7	Daily film clip or scientific input	Behavioral activation task 7	Dashboard
8	Reflecting about strengths and resources using the tree of resources^d^ [29]	Participants write down individual resources inside their tree of resources in order to visualize and reflect about personal strengths and positive aspects of life.	Implementation intention 8	Daily film clip or scientific input	Behavioral activation task 8	Dashboard
9	Thinking about the desired personality using miracle questions^a^ [16]	Miracle questions are thought experiments, which ask people to imagine their desired personality, their desired future and specific plans and their priorities for the next five years.	Implementation intention 9	Daily film clip or scientific input	Behavioral activation task 9	Dashboard
10	Looking forward and thinking about the future^d^ [30]	Participants should think about future plans, dreams, hopes, and poss	Implementation intention 10	Daily film clip or scientific input	Behavioral activation task 10	Dashboard

*Note.* ^a^Actuating discrepancy awareness; ^b^targeting thoughts and feelings to realize insight; ^c^targeting behaviors to realize practice; ^d^activate strengths and resources to realize strengths-orientation; since these general change mechanisms are overlapping in content, weekly core themes and micro-interventions might fit to more than just one general change mechanism

### Weekly Core Themes and Micro-Interventions

The structure of the PEACH intervention includes (a) weekly core themes with specific micro-interventions and (b) micro-interventions that are not directly related to the weekly core themes. The weekly core themes and the micro-interventions that were used every day for 10 weeks are shown in Table 1. In the following, we briefly discuss the six types of micro-interventions that were used in PEACH: (1) individualized implementation intention, (2) psychoeducation, (3) behavioral activation, (4) self-reflection, (5) resource activation, and (6) individualized progress feedback. The included micro-interventions were selected to target and to activate the general (common) change mechanisms in order to maximize the effects of the intervention [7].

#### Individualized implementation intentions

An implementation intention is a self-regulatory strategy in the form of an “if-then plan” that can lead to better goal attainment [5, 24]. This micro-intervention targets the general change mechanism *targeting behaviors to realize practice.* Participants generate one individual and specific implementation intention based on suggested behavioral activation task every Sunday. This individually built implementation intention should be implemented in daily life during the following week as often as possible. Examples for implementation intentions are: “If I have to work concentrated, then I switch into flight mode” (Productivity, Conscientiousness), “If I have no meetings before 1:00 p. m,, then I will go to the gym.” (Productivity, Conscientiousness) or “If I see something beautiful, then I will take a photo.” (Aesthetic Sensitivity, Open-Mindedness).

#### Psychoeducation

Psychoeducation fosters knowledge transfer about personality dispositions, personality change and its outcomes. This micro-intervention operationalizes the general change mechanism *targeting thoughts and feelings to realize insight.* In the present coaching intervention, participants receive every morning either a short film clip or a message with scientific “food for thought”. In total, we developed 36 film clips (11 film clips providing information about personality dispositions and personality change in general and 5 film clips for each participant fitting to the chosen change goal and its outcomes) and 104 scientific messages (34 providing input about personality dispositions and personality change in general and 10 messages for each participant fitting to the chosen change goal). Film clips provide worst- and best-case scenarios and scientific facts about the advantages of achieving the desired change. These interactive elements should also promote motivation and adherence among participants.

#### Behavioral activation tasks

Behavioral activation directly changes actual behavior and reinforces new behavior. This micro-intervention operationalizes the general change mechanism *targeting behaviors to realize practice.* In the present coaching intervention, participants receive three new suggestions of behavioral activation tasks every Sunday, which fit to their chosen change goal [25, 26]. Out of these three suggestions, participants select one behavioral activation task with the goal to implement the task in their daily routine during the following week. Examples for behavioral activation tasks are: “Don’t procrastinate and do things right away.” (Productiveness, Conscientiousness), “Tidy up a part of your flat every day.” (Organization, Conscientiousness) or “Take a photo of something beautiful every day.” (Aesthetic Sensitivity, Open-Mindedness). In total, we developed 12 behavioral activation tasks for each of the nine Big Five personality trait profiles (108 behavioral activation tasks in total) (cf. [59]).

#### Self-reflection

Self-reflection is a tool to exercise introspection, learn from experiences including successes and failures. This micro-intervention is included to target the general change mechanism *targeting thoughts and feelings to realize insight.* Different tools to exercise self-reflection are included in the weekly core themes, which change every week to enhance adherence and are embedded in every dialogue in the evening (Table 1).

#### Resource activation

Resource activation capitalizes on individual and interpersonal strengths and resources***.*** This micro-intervention is included to target the general change mechanism *activating strengths and resources to realize strengths-orientation*. Tools including resource activation are also included in the weekly core themes, which change every week (Table 1).

#### Individualized progress feedback

Individually tailored progress feedback is one of the most commonly used change techniques in smartphone-based health interventions [13] that helps people to focus on their discrepancy awareness. This micro-intervention targets the general change mechanism *actuating discrepancy awareness.* Participants constantly receive individualized graphical feedback on the dashboard of the PEACH app (Fig. 2). For instance, they can check whether they are already approaching their change goal compared to the beginning of the intervention. Additionally, they get feedback about how often they had opportunities to show their weekly implementation intention and how often they actually implemented it during the last seven days. Furthermore, they can check their momentary status (bronze, silver or gold status) and see the credits they have already earned during the intervention (Fig. 2).

### Assessment Strategy

The assessment strategy includes (1) a screening assessment (self-reported), (2) an outcome assessment (self-reported and observer-reported), (3) a process assessment (self-reported), and (4) smartphone sensing. An overview is shown in Table 2. These different types of assessments will be further elaborated in the following.

**Table 2:** Measures

				Intervention				
	Screening	Pre-Test	Experience Sampling	Daily	Weekly	Post-Test	Follow-up	Evaluation
Screening								
Symptom-Check List (SCL-K11; [62])	x							
Depression Scale (ADS-K; [63])	x							
Demographics	x	x						
Main Outcome Assessment – Self report								
Big Five Personality Inventory (BFI-2; [59])		x				x	x	
Main Outcome Assessment – Observer Report								
Big Five Personality Inventory (BFI-2-S; [61])		x				x	x	
Process Assessment – Self report								
Big Five Personality Inventory 2 (BFI-2-S; [61])					x			
Big Five personality states			x	x				
Affect (PAM; [75])			x	x				
Information about current environment			x					
Stress level			x					
Realization of implementation intention				x				
Opportunities for realization of implementation intention				x				
Strength of change goal		x			x	x	x	
Subjective perception of change					x	x	x	
Learning experience					x			
Inclusion-of-the-Other-in-the-Self [71]		x			biweekly	x		
Working Alliance Inventory (WAI-SR, [67])		x			biweekly	x		
Perception of Robots [69]		x				x		
Trust [70]		x			four-weekly	x		
Further Outcome & Control variables – Observer report								
Demographics		x				x	x	
Type and closeness of relationship		x				x	x	
Time spent with target person		x				x	x	
Further Outcome & Control variables – Self report								
Willingness to change [60]		x						
Implicit theory of personality [64]		x				x	x	
Satisfaction with life domains [2]		x				x	x	
Satisfaction with Life Scale [65]		x				x	x	
Rosenberg Self-Esteem Scale (RSES; [66])		x				x	x	
Engagement in self reflection		x				x		
Engagement in practice		x				x		
Feedback on components of the coaching								x
Technology acceptance scales [74]		x						x
Internet users’ privacy concerns [89]		x						
Technical anxiety [90]		x						
Manipulation check items		x						x

#### Screening assessment

During the onboarding process (Fig. 1), participants will respond to two screening questionnaires to check for eligibility. Participants are directed from the PEACH app to the online survey tool (limesurvey.org), so that they can answer the screening questionnaires on their smartphone. Short forms of the Symptom-Check List (SCL-K11; [62]) and Depression Scale (ADS-K; [63]) will be used to assess mental health disorders and other psychosocial problems (Table 2). Individuals with scores above the cut-off value in the SCL-K11 (≥14) and above the cut-off value in the ADS-K (≥19) will be excluded and are provided with information and contact details of the psychological counseling service of the University of Zurich.

#### Outcome assessment

##### Self-reports

**Fig. 3:**
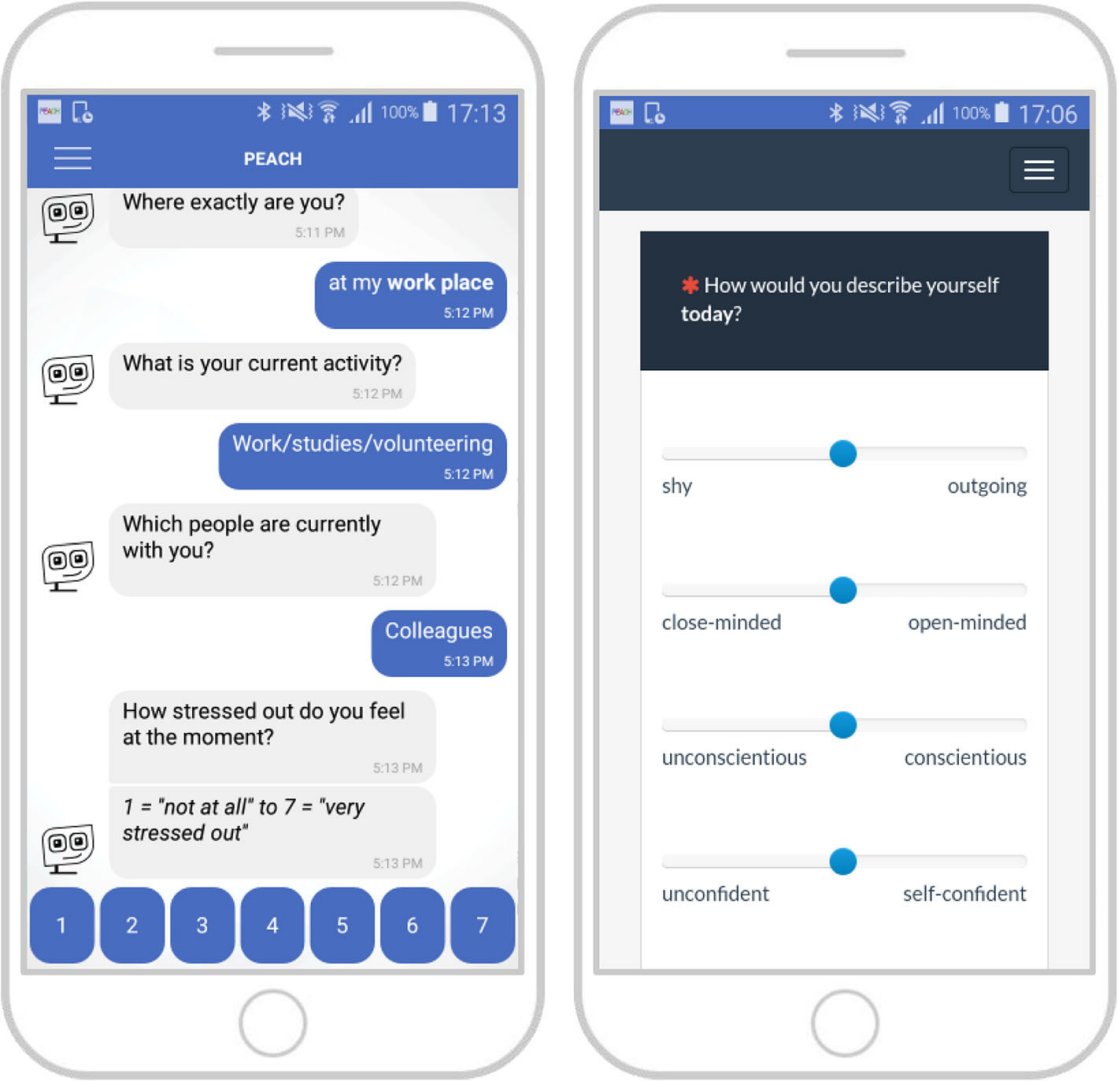
User interface for survey data collection. *Note.* Experience sampling assessment with self-reports (left) and daily diary assessment of the Big Five personality states (right) using bipolar adjective items (ad hoc translation from German)

The self-reports include a pre-test, a post-test and a three-month follow-up assessment. Pre-test assessment will take place before the intervention, post-test assessment after the intervention and the follow-up assessment three months after the end of the intervention to check whether personality changes could be maintained over a longer period of time or revert over time. At all points of measurement participants will be automatically directed from the PEACH app to the online survey tool (limesurvey.org) to answer all questionnaires on the smartphone (Fig. 3). The main outcome assessment includes the Big Five Inventory 2 (BFI-2; [59]) to assess the Big Five personality traits and trait-related facets. Further outcome variables and control variables are willingness to change [60], implicit theory of personality [64], satisfaction with life (SWLS; [65]), satisfaction with life domains [2], and self-esteem (RSES; [66]) (Table 2).

Main outcomes regarding the relationship building process with the conversational agent include the following: Working alliance will be assessed using an adapted short-version [67] of the Working Alliance Inventory (WAI-SR) [52] based on Kiluk et al.’s [68] work, who adapted the complete WAI to measure working alliance with a technology-based intervention (WAI-Tech). To further understand the perception of the conversational agents, the Perception of Robots scale [69] and trust measures [70] to assess trust development mechanisms will be included. Interpersonal closeness will be measured with the Inclusion-of-the-Other-in-the-Self (IOS) scale [71], an established and reliable instrument to measure perceived closeness of a relationship [72]. Manipulation checks for perceived self-awareness of the conversational agents [73] will be conducted every four weeks to confirm that the manipulation had been scripted thoroughly throughout the 10-weeks of intervention.

Overall satisfaction with the app, ease of use, perceived enjoyment, and perceived usefulness will be measured after the first week and at the end of the intervention [74] to identify differences in the perception of the conversational agent due to differences in the usability of the app. Moreover, we are interested in qualitative feedback of users at the beginning of the interaction with the conversational agents (first impression) and at the end of the intervention to eventually improve the interaction with and perception of the conversational agents.

##### Observer reports

In addition to self-reports, observer-reports by close others will be assessed. At the beginning of the study, participants will be asked to share a link to the online observer-report questionnaires with at least three close friends, family members or their intimate partner. Observer-reports include the Big Five personality traits (BFI-2-S; [61]), type and closeness of the relationship and time spent with the target person. Observer-reports will be assessed at pre-test, post-test and follow-up assessment (Table 2).

#### Process assessment

##### Experience sampling with self-reports

One week before the intervention and one week after the intervention (Fig. 1), there will be an experience sampling with self-reports including four assessments per day (Fig. 3) at random times once in each of four predefined time windows: 9:30 a. m. - 11:30 a. m., 12:30 p. m. - 14:30 p. m., 15:30 p. m. - 17:30 p. m., 18:30 p. m. - 20:30 p. m. Participants will be asked to behave the first week as normal as possible and not to change anything in their behavior in order to measure their baseline behavioral signatures. After the intervention, participants are asked to answer the same experience sampling questions again during one week. This allows to check for changes in the behavioral signatures as a result of the intervention. Experience sampling assessments include the photographic affect meter (PAM; [75]) to assess momentary affect, five bipolar adjective items to assess the Big Five personality states (presentation in a random order), a few questions about the current location (e.g., indoors versus outdoors), activity, and social environment (e.g. alone versus with other people) at the moment, and a single item to measure momentary stress.

##### Daily diary and weekly self-report assessments

During the intervention, there will be daily diary and weekly measurements to assess individual change progress. The photographic affect meter (PAM; [75]) and ten bipolar adjective items to assess Big Five personality states will be used on a daily basis every evening. Additionally, participants are asked every evening whether they had opportunities to show their individual implementation intention and whether they could perform their implementation intention. There will be a weekly assessment every Sunday including a short version of the BFI-2 (BFI-2-S; [61]) (Table 2).

#### Smartphone sensing

Smartphone applications can get access to data from sensors and usage logs (e.g. location, surrounding devices via Bluetooth, logs of application usage and phone calls), which allow objective measurement of behavior, and inferences about users’ personality [76, 77]. Using this data, it may also be possible to detect changes in personality over short periods of time. If the application detects changes in behavior that are consistent with desired changes in personality and associated with the use of the PEACH app, this would constitute complementary evidence for PEACH’s effectiveness.

### Technological Background of the Intervention

The smartphone-based coaching intervention is based on the MobileCoach (www.mobile-coach.eu). The MobileCoach is an open source platform for the design, delivery and evaluation of scalable smartphone-based interventions [40, 43, 78]. It is available via the research and industry-friendly Apache 2 license and follows a client-server model. The rule-based intervention logic and messages are defined by intervention authors on the server. MobileCoach then acts as a conversational agent and uses these rules to send out the intervention messages to client applications on mobile devices. MobileCoach also allows to react to answers given by intervention participants and can deliver these interventions via the widely available short message service (e.g., to lower the threshold for participation and to maximize the reach of an intervention) and/or via dedicated mobile messaging apps for Apple’s iOS and Google’s Android platform. The mobile messaging apps allow not only to fully customize the user experience to a particular target group (e.g., the look and feel of the app with various conversational agents) but also to use sensor data from smartphones and/or other connected devices to deliver just in time adaptive interventions based on end users’ states of receptivity and vulnerability [79, 80].

Previous studies have demonstrated the effectiveness and reach of MobileCoach-based interventions with regard to problem drinking in adolescents [58] and perceived ease of use, enjoyment, therapy adherence and scalability in a childhood obesity intervention [40, 43]. In addition to delivering interventions, MobileCoach is also used for the collection of intensive longitudinal data in situ (e.g., for ecological momentary assessments), for example in a clinical trial on stress disorders.

In the PEACH study, the iOS and Android apps of MobileCoach are used to guide participants through the micro-interventions, providing motivation, promoting commitment, and reminding them to complete questionnaires. During these conversations, a combination of pre-defined answers and free-text input is used to constrain the dialog along pre-defined counselling paths and to give the participant autonomy where needed (e.g., for the definition of implementation intentions in the if-then form). With a swipe-to-the-right gesture or via a menu button, participants can open the sidebar of the PEACH app (Figure 2) from which they can navigate to (a) their personal dashboard, (b) a media library used for psychoeducation video clips which are unlocked along the intervention path, (c) a second chat channel “Support-Team” for a traditional WhatsApp like communication with the study team (e.g., to clarify technical questions and comments), or (d) to a page for frequently asked questions about the PEACH study and the PEACH app. From the sidebar, participants can also navigate back to the chat channel with the conversational agent and to the dashboard (Fig. 2). The dashboard gives an overview of the self-selected personality change goal and the weekly individual implementation intention. It also provides a traffic light, whether an individual was able to get closer to its intended personality change goal (green light), further away from it (red light) or whether there is no change in any direction (yellow light). For this comparison, participants self-reported ratings of the Big Five personality states (bipolar adjective scales) for the last seven days will be compared to his/her ratings of the Big Five personality states of the first week of the intervention. The traffic light changes to green or red when the averaged delta of scores between the last and the first week is more than half a standard deviation. The rationale of using a time window of seven days was to account for natural variance in daily ratings of study participants. The dashboard also visualizes whether and on which day participants had opportunities to pursue and in fact realize their individual implementation intention during the last seven days. Eventually, the personal dashboard illustrates their latest credit score and the remaining time of the intervention program.

### Data Analyses

Longitudinal multilevel modeling (MLM) and structural equation modeling (SEM) will be used to analyze the (intensive) longitudinal, nested data structure and change over time [81–84]. Both data-analytic methods are specifically suitable to model change explicitly as a function of time and can be used to formulate equivalent models, providing identical estimates for the collected data. Separate models will be analyzed including the outcome assessments at pre-, post- and follow-up (self-reports and observer-reports), daily diary assessments, and weekly self-report assessments. Predictor/control variables will be added to the models to examine how individual growth will be moderated by variables such as intervention condition, change goal or willingness to change. The statistical modeling programs Mplus [85], and updated R packages (R Core Team, Vienna Austria) will be used to estimate the growth curve models.

## Discussion

This study is the first one testing the effectiveness of a smartphone- and conversational agent-based theory-driven intervention for intended personality change to support people who want to change self-selected personality traits. Understanding short-term changeability of personality traits in daily life and examining whether potential intentional personality trait changes can be maintained or rather revert over time is a key goal in the research fields of personality development and personality dynamics and complements previous longstanding work on long-term changes of personality traits across the lifespan. This is particularly important because personality changes can have a powerful impact on people’s lives. For example, becoming more conscientious over longer time periods is related to more health-related behaviors and ultimately to better health and well-being [86, 87]. Intended personality changes such as decreases in neuroticism may also reduce economic costs [88].

The study will not only advance our understanding of the short-term changeability of personality traits and intended efforts to change them, but also increase our knowledge of underlying short-term processes and dynamics of change. Furthermore, the study contributes to the understanding of the design of text-based conversational agents and the role of conversational agents in supporting and coaching individuals to reach their individual change goals. This is particularly interesting since empirical evidence of text-based conversational agents on the effectiveness of smartphone-based interventions is still sparse. The application of the talk-and-tools paradigm used in the PEACH mobile app can not only complement and extend existing face-to-face counseling sessions to the everyday life of individuals, but can also provide new means to offer smartphone-based coaching interventions in a scalable fashion where a personal coaching approach is not feasible due to limited reach, personnel or budget.

Given that the intervention approach of the PEACH mobile app proves effective, it could be easily implemented in various non-clinical settings such as counseling/mentoring (e.g., individual change processes) or coaching (e.g., personality related aspects in diet, fitness, health and well-being) and could reach large numbers of people due to its low-threshold character.
